# PTEN Deletion in Adult Mice Induces Hypoinsulinemia With Concomitant Low Glucose Levels

**DOI:** 10.3389/fendo.2022.850214

**Published:** 2022-02-25

**Authors:** Maria Crespo-Masip, Aurora Pérez-Gómez, Carla Guzmán, Sandra Rayego, Nuria Doladé, Alicia García-Carrasco, Ramiro Jover, José Manuel Valdivielso

**Affiliations:** ^1^ Vascular & Renal Translational Research Group, IRBLleida, Spain and Spanish Research Network for Renal Diseases (RedInRen. ISCIII), Lleida, Spain; ^2^ Experimental Hepatology Unit, IIS Hospital La Fe, Valencia, Spain; ^3^ CIBERehd, Centro de Investigación Biomédica en Red de Enfermedades Hepáticas y Digestivas, Instituto de Salut Carlos III, Madrid, Spain; ^4^ Department Biochemistry and Molecular Biology, University of Valencia, Valencia, Spain

**Keywords:** PI3K/AKT, hypoinsulinemic hypoglycemia, gluconeogenesis, lipid metabolism, glucose metabolism

## Abstract

The PI3K/AKT pathway, negatively regulated by PTEN, plays a paramount role in glucose metabolism regulation due to its activation by the insulin receptor signaling pathway. We generated a PTEN-KO mouse to evaluate the systemic effect of the overactivation of the PI3K/AKT pathway in insulin signaling and glucose homeostasis. Our results demonstrate that PTEN-KO mice show very low glucose levels in the fasted state, which poorly respond to glucose and pyruvate administration. Insulinemia decreased without alterations in pancreatic islets. Among the possible reasons, we uncover the deregulation of the expression of proximal tubule glucose transporter and consequent glycosuria. Moreover, we evidence an altered activation of hepatic gluconeogenesis-related genes. In addition, the expression of several genes related to β-oxidation showed a delayed or even absent response to fasting, suggesting that the lack of PTEN not only impairs glucose metabolism but also slows down the use of lipids as a metabolic fuel. We conclude that the inducible full PTEN-KO mice could be a good model to study the metabolic interactions between glycidic and lipidic metabolism in hypoinsulinemic hypoglycemia and that PTEN could be an important mediator in the disease and/or a potential drug target.

## Introduction

Hypoglycemia can be induced by many causes, especially by an overdose of oral hypoglycemic agents or insulin in diabetic patients, leading to hyperinsulinemic hypoglycemia. However, in rare cases, hypoglycemia can be found together with low levels of insulin in non-diabetic patients, in the so-called hypoinsulinemic hypoglycemia. Liver injury-induced hypoglycemia is one of the causes of hypoinsulinemic hypoglycemia (1). In those cases, hypoglycemia seems to be secondary to a depletion of glycogen in the liver. Hypoinsulinemic hypoglycemia has been also reported in Doege-Potter syndrome, a rare paraneoplastic condition characterized by a solitary fibrous tumor. This tumor causes hypoglycemia by the secretion of a prohormone form of insulin-like growth factor II (IGFII) (2). Big IGFII, the high‐molecular-weight isoform of pro‐IGFII, causes hypoglycemia by several mechanisms, but most are thought to be mediated by stimulation of the insulin receptors (IR) (3, 4). Other potential but less common causes of hypoinsulinemic hypoglycemia include the production of autoantibodies against the insulin receptor and mutations in protein kinase B (PKB), also known as serine/threonine kinase AKT (5–7). Hypoglycemia can lead to several symptoms like weakness, sweating, followed by confusion/disorientation and shakiness (8). In hypoinsulinemic hypoglycemia, episodes of hypoglycemia are usually recurrent and refractory to conventional treatment, and they can cause life-threatening consequences (9, 10).

Phosphatase and tensin homolog (PTEN) is a non-redundant dual phosphatase that negatively regulates the PI3K/AKT pathway, controlling essential processes to maintain the cell homeostasis (growth and metabolism, cell polarity and migration, cell-cycle progression, stem cell renewal, and cell architecture and environment) (11–13). The PI3K/AKT pathway plays a paramount role in glucose metabolism regulation. Insulin binding to the IR located in target cells will lead to the activation of phosphatidylinositol 3-kinase (PI3K) which is antagonized by PTEN. Subsequent AKT stimulation activates glycogen synthase to help store the glucose as glycogen in hepatocytes, decreasing blood glucose levels (14, 15). AKT is also involved in the inhibition of gluconeogenesis in the liver by phosphorylating some gluconeogenesis-related transcription factors like Forkhead box O1 (FOXO1). Then, the phosphorylated FOXO1 will be excluded from the nucleus and consequently, the expression of FOXO1 target genes such as the key gluconeogenesis genes glucose-6-phosphatase catalytic subunit (G6PC) and phosphoenolpyruvate carboxykinase (PEPCK) will be down-regulated. In addition, it is known that AKT phosphorylates peroxisome proliferator-activated receptor-gamma coactivator 1 alpha (PGC1a) mediating the transcriptional repression of G6PC and PEPCK genes, as well as other genes involved in fatty acid oxidation (16, 17). This regulation is essential to control the response of metabolic organs like the liver, muscle and adipose tissue to high insulin.

The investigation of hypoinsulinemic hypoglycemia is hampered by the lack of animal models to study its effects and possible targets to design efficient treatments. In the present study, we generate an inducible full PTEN knock-out model (PTEN-KO) to evaluate the systemic effect of the overactivation of the PI3K/AKT pathway in insulin signaling and glucose homeostasis and to assess its suitability as a hypoinsulinemic hypoglycemic mouse model.

## Materials and Methods

### Ethical Statement

All animal procedures were approved by the University of Lleida Animal Ethics Committee and followed the guidelines of the European Research Council and local laws for the care and use of laboratory animals. In all surgical procedures performed in animals, isoflurane was used as anesthetic. Animals were kept in a 12-hour light-dark cycle at 22°C with *ad libitum* access to regular mouse chow (Teklad Global 14% Protein 4% Fat Rodent Maintenance Diet – Envigo. Harlan Teklad, Madison, WI; USA) and water unless otherwise specified.

### Tamoxifen Inducible PTEN-KO Mouse Model

Cre-Estrogen receptor induced by tamoxifen (Cre-ER^TM^ )[B6.Cg-Tg(CAG-Cre/Esr1* 5Amc/J] and floxed homozygous PTEN (*C;129S4-Ptentm1Hwu/J*; hereafter PTEN-KO) mice were donated by Dr. Xavier Dolcet (University of Lleida, Spain) after being acquired from the Jackson Laboratory (Bar Harbor, ME) (18). Mice were weaned and genotyped as previously described at 21 days after birth (19, 20), (**Supplementary Figure 1** and **Supplementary Table 3**). Tamoxifen (Sigma-Aldrich T5648, St Louis, MO) was dissolved in absolute ethanol and diluted in corn oil (Sigma-Aldrich C8267) to a final concentration of 5 mg/ml. To induce PTEN ablation, a single intraperitoneal injection of 25mg/kg of tamoxifen was administered to 4-5 weeks old mice as previously described (18). All the experimental groups were formed by male and female animals in an approximate 50% proportion.

### *In Vivo* Experiments

Two months after the tamoxifen injection, the experiments were performed. Some animals were sacrificed by terminal blood collection and organs were harvested after perfusion with saline solution through the left ventricle. One part of the tissue was snap-frozen, while the other was fixed in 4% paraformaldehyde for histological assessments.

In some animals, blood glucose fluctuations were measured at several time points up to 420 minutes after food removal. We also performed a glucose tolerance test (GTT) in mice previously fasted for 2h. Then, an intraperitoneal single injection of 4g/kg of glucose (Sigma-Aldrich G8270) was administered. Blood glucose measurements were taken at times 20, 40, 60, and 120 minutes with a glucometer (Accu-Check Performa – Roche, Basel, Switzerland). In some of the animals, 20uCi of ^3^H glucose (Perkin-Elmer NEC042X250UC) was added to the glucose injection, to determine the organ-specific uptake of glucose. At the end of the GTT, several organs were collected, and 100 mg of tissue were homogenized in 1 ml of distilled water. 800 ul of the mixture were centrifuged and 25 ul of the supernatant were mixed with 10 ml of scintillation fluid to determine total ^3^H radioactivity. Results were normalized by ^3^H counts present in 200ul of blood of each animal. For the pyruvate tolerance test (PTT), the same protocol of the GTT was followed, replacing glucose with a dose of 2g/kg of sodium pyruvate (Gibco by Life Technologies - 11360-070). In order to detect glucose in urine, the glucose quantitative determination kit Spinreact-Glucose-TR 1001190 was used.

Total cholesterol (TC), HDL-C and triglycerides (TG) were measured by colorimetric methods according to standardized protocols with an AU5800 Analyzer (Beckman Coulter Inc, Fullerton, CA, USA) in the Clinical Analysis Laboratory of Arnau de Vilanova University Hospital, in Lleida, Spain. LDL-C was calculated by the Friedewald equation if TG <250 mg/dL or by a colorimetric method if TG >250 mg/dL. Blood Urea Nitrogen (BUN) was determined by colorimetric assay using the QuantiChrom Urea assay kit (DIUR-500 of BioAssay Systems, Gentaur, San Jose, CA, USA). To detect serum ketone body levels the Sigma-Aldrich MAK041 β-hydroxybutyrate assay kit was used. Insulin and C-peptide levels in blood were determined with the Rat/Mouse ELISA for insulin (EZRMI-13K - EMD Millipore) and the C-Peptide 2 Elisa kit (EZRMCP2-21K - EMD Millipore). The protocols used were the ones supplied by the manufacturer.

### Histopathology and Immunohistochemical Analysis

Paraffin blocks were sliced at 5 μm, dried for 30 minutes at 60°C, and then followed a process of dewaxing and rehydration before being stained with hematoxylin (PanReac AppliChem ITW Reagents 251344) and eosin (Master diagnostic MAD-109 1000). Finally, samples were dehydrated and mounted with mounting medium. For the periodic acid Schiff- alcian blue (PAS-AB) staining after rehydration, slides were incubated 5 minutes with AB, 15 minutes with periodic acid, and then, 25 minutes in the Schiff solution (4g basic fuchsin + 7,6g sodium metabisulfite + 400ml hydrochloric acid 0,25N).

For the immunohistochemistry staining (IHC), rehydrated samples were treated with antigen retrieval solution (10mM citrate buffer, pH6) for 30 minutes at 100°C. Next, the blockage of endogenous peroxidase was performed with blocking solution (200 ml PBS1x + 4 ml peroxide hydrogen) for 30 minutes followed by antibody non-specific binding blocking (Vector Laboratories, VECTASTAIN® Elite ABC-HRP Kit (Peroxidase, Universal) PK-6200) for 30 minutes at room temperature. The primary antibody used was anti-insulin (Polyclonal Guinea Pig Anti-Insulin A0564, Dako, Denmark. 1/100 dilution) and incubated overnight at 4°C. The secondary antibody used was VECTASTAIN® Elite ABC-HRP Kit (Peroxidase, Universal. 1/50 dilution). Finally, the reaction was visualized with DAB chromogen (DAB peroxidase substrate kit, Vector Laboratories) for 10 minutes at room temperature.

For the pancreatic islet area determination, serial sections of the pancreas stained with H&E were examined. The section with the higher diameter for each islet was selected. In that section, the area of the islet was determined by ImageJ software. Same procedure was used to establish the area of the pancreas. The added area of the islets was then referred to as the maximum area of the pancreas. Insulin-stained area was measured by a similar procedure in sections with an IHC for insulin.

### Pancreatic Islet Experiment

Pancreatic islets were isolated as previously described by Carter et al. (21) using 4 ml of 0.8mg/ml collagenase (Collagenase from Clostridium histolyticum C6885-500mg - Sigma). Next, *ex vivo* incubation of pancreatic islets was performed following the Nolan and O’Dowd (22) protocol in 15mM glucose. After 1 hour of incubation, supernatants were collected, and insulin secretion was measured.

### Hepatic Glycogen Detection

We placed 100mg of liver tissue in 500μl of homogenization buffer (50mM TrisHCl pH 7.5 + 1.86g EDTA (5mM) + 0.15g DTT (1mM) +10μl/ml PMSF + 5μl/ml PIC + 5μl/ml Na_3_VO_4_) together with stainless steel beads. The tube was inserted into the TissueLyser LT (Qiagen) machine for one minute at 50Hz. Then, the liver homogenate was mixed with 100μl of Tris 50mM buffer and 100μl of perchloric acid 0.2M and centrifuged. The supernatant was transferred to a new tube with 300μl of ethanol 90% and stored at -20°C overnight. Then, samples were centrifuged to precipitate the glycogen pellets, which were let to dry completely. The dried glycogen pellets were mechanically resuspended in 2ml of 2M HCl. After 20 minutes at 100°C, 1ml of NaOH 4M and 1ml of 1% 3,5-dinitrosalicylic acid (DNS) were added to both experimental and standard curve samples. Finally, the tubes were boiled at 100°C for 5 minutes and their absorbance was read at 546nm.

### *In Vitro* Experiments

Human kidney-2 cells (HK-2), an immortalized cell line from proximal tubule cells of adult human kidneys, were cultured as previously described in our laboratory (23). The protocol of lentiviral production and infection was followed as previously described (23). The empty vector used as a control in this study is a modification of fsv-si (9427bp) containing the Venus variant of GFP under the control of the SV40 promoter. The PTEN silencing sequence was GCAGTATAGAGCGTGCAGATA. The efficiency of infection was determined by fluorescence detection of the GFP protein. Cultures with an efficiency of 90-100% were used.

### Quantitative Reverse Transcription PCR (qRT-PCR)

To extract mRNA, 1ml of trizol was added to the culture plate and the cell monolayer was scratched. In kidney tissue samples, disruption was performed in 300μl trizol with stainless steel beads and 3 cycles of 30 seconds at 50 Hz with the TissueLyser. Then, 200μl of chloroform was added, mixed, and centrifuged. The aqueous phase was transferred to a second tube and 500μl of isopropanol was added and incubated for 10 minutes. After centrifugation and precipitation of RNA, the pellet was washed with 1 ml of ethanol 75%. Samples were dried for 15 minutes and 50μl of nuclease-free water (Sigma-Aldrich 3098) was added to resuspend the RNA. Liver mRNA was extracted from 20mg of tissue with the RNA isolation kit (NucleoSpin RNA Macherey-Nagel 740955) after following the protocol detailed on the kit. The mRNA concentration was calculated with the NanoDrop spectrophotometer (ND-1000 Spectophotometer) and frozen at -80°C.

The reverse transcription reaction was made with 9.5μl of 0.1μg/μl mRNA sample and 10.5μl of the retrotranscription mixture (2μl Reaction Buffer 10x, 4μl MgCL2 25mM, 2μl dNTPs, 2μl Random Hexamers and 0.5μl AMV Reverse Transcriptase). Then, the qPCR reaction was performed as previously described (23). The primers used can be found in **Supplementary Table 1**.

### Western Blot Analysis

For the protein extraction from cell cultures 200μl/well of lysis buffer (Tris pH=7.5 20mM, 2% SDS) was added to the plate and the cell monolayer was scratched. Then, cells were sonicated and centrifuged. Tissue protein extraction was done with 300μl of lysis buffer (20mM Tris/HCl pH=7.5, 120mM NaCl, 0.5% Igepal CA-630 (Nonidet P-40), 100mM NaF, 10μl/ml of 0.1M PMSF, 5μl/ml of X M PIC; 5μl/ml of 0.2M Na_3_VO_4_) added to tissues. Then, a 1-minute cycle at 50Hz with 7mm stainless steel beads in the TissueLyser was performed. The protocol used for western blot has been previously described (23). The antibodies used can be found in **Supplementary Table 2**.

### Statistical Analyses

All experiments were performed at least three times. Statistical analyses were performed with GraphPad Prism 7.0 software. Values are presented as mean ± standard error of the mean (SEM). Data were compared by using the Student’s t-test or by two-way ANOVA followed by Bonferroni’s test for multiple comparisons, with p<0.05 considered to be significant.

## Results

### PTEN-KO Mice Exhibit Low Blood Glucose Levels and Respond Poorly to Glucose and Pyruvate

**Figure 1:**
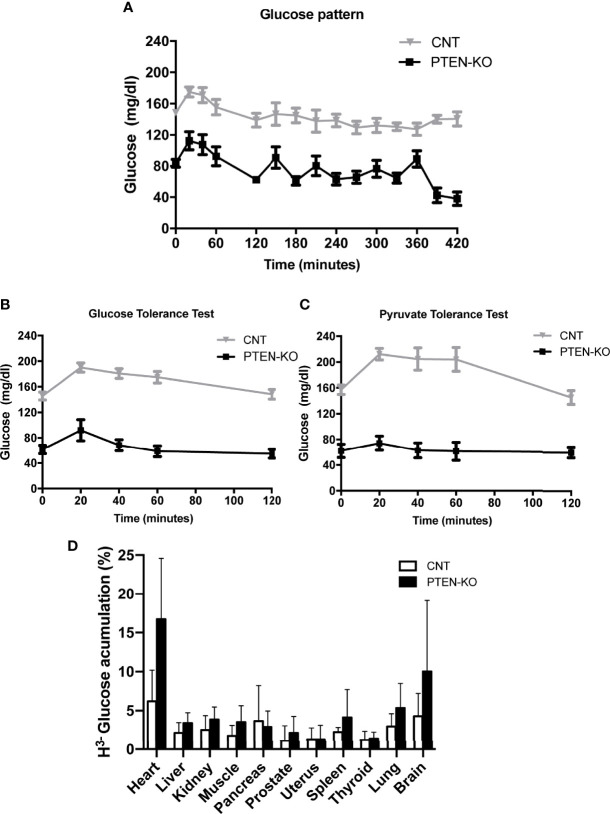
Blood glucose behavior is altered in inducible PTEN-KO mice. **(A)** Time course of blood glucose levels in mice after fasting. **(B)** Glucose tolerance test. **(C)** Pyruvate tolerance test. **(D)** ^3^H-glucose uptake test. Data represent the mean ± SEM of 15-20 mice/group **(A, B)**, 8-11 mice/group **(C)** and 7 mice/group **(D)**. Two-way ANOVA test performed in 1D showed a significant result for the genotype comparison (CONTROL *vs* PTEN-KO).

The first remarkable finding was the low serum glucose level of PTEN-KO mice in the basal time point (two months after PTEN ablation, time 0), as well as after 2h and 7h of fasting, compared to control mice (CNT) (**Figure 1A**). In addition, after 7 hours of fasting, the glucose levels of control mice were similar to their basal levels, in contrast to PTEN-KO mice in which blood glucose levels were significantly lower than the ones measured at the basal time point. Furthermore, the GTT showed that PTEN-KO mice responded with a smaller peak in serum glucose, which returned to basal values after 40 minutes, in contrast to control mice in which glucose levels returned to basal values after 120 minutes (**Figure 1B**). Moreover, the PTT showed that plasma glucose levels were not modified by pyruvate injection in PTEN-KO mice in contrast to CNT mice in which glucose levels increased after 20 minutes (**Figure 1C**). Most organs of the PTEN KO mice showed a tendency to an increased uptake of ^3^H-glucose. Although no differences between different organs were found (**Figure 1D**), two-way ANOVA analysis showed a statistically significant difference between both phenotypes. Food consumption was higher in PTEN-KO mice (**Supplementary Figure 2A**), although it did not lead to differences in body weight with respect to CNT mice (**Supplementary Figure 2C**). PTEN-KO mice showed an almost total absence of body fat (**Supplementary Figure 2D**). No significant differences were observed in water intake (**Supplementary Figure 2B**).

### PTEN-KO Mice Have Low Insulinemia With Preserved Insulin Secretion Capacity

**Figure 2:**
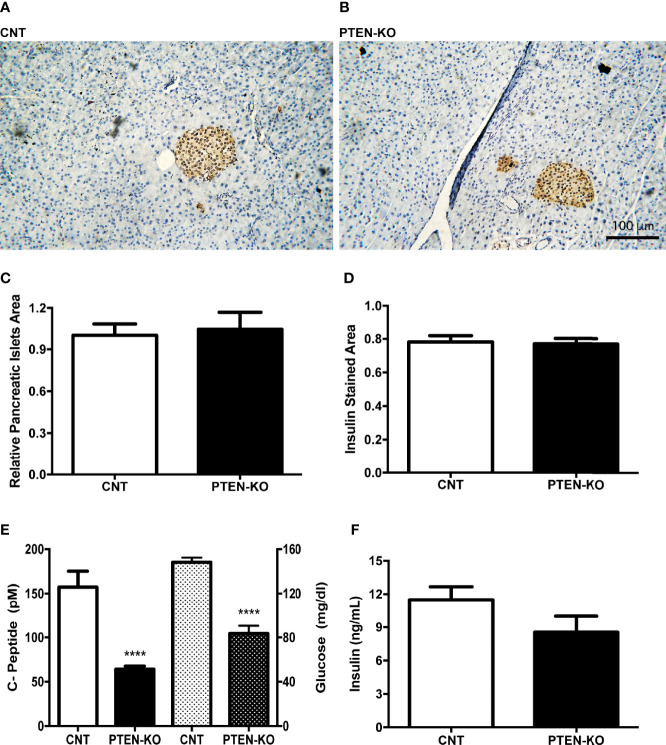
Normal pancreatic islets and reduced insulin levels in serum of inducible PTEN-KO mice. **(A, B)** Representative pictures of pancreatic islets stained with insulin antibody. **(C)** Relative pancreatic islets area. **(D)** Insulin-stained area related to total pancreatic islets area. **(E)** C-peptide (solid bars) and glucose (dotted bars) levels in serum. **(F)** *Ex vivo* insulin secretion from incubated pancreatic islets. Original magnification x20 **(A, B)** Data are mean ± SEM of 5 mice/group **(A–D)** and 21-23 mice/group **(E)**. Data represent the mean ± SEM from 3-5 independent experiments **(F)**. ****p < 0.001 *vs*. CNT mice.

C-peptide levels in serum of PTEN-KO mice were statistically significantly lower compared to CNT mice, as well as the glucose levels (**Figure 2E**). However, the relative pancreatic islet area and the insulin-stained area in PTEN-KO mice were not different from control mice (**Figures 2A–D**). In addition, *ex vivo* incubation of pancreatic islets showed no differences in the insulin-secreting capacity in response to glucose (**Figure 2F**).

### Slight Glycosuria and Kidney Glucose Transporters Alterations in PTEN-KO Mice With Normal Renal Function

**Figure 3:**
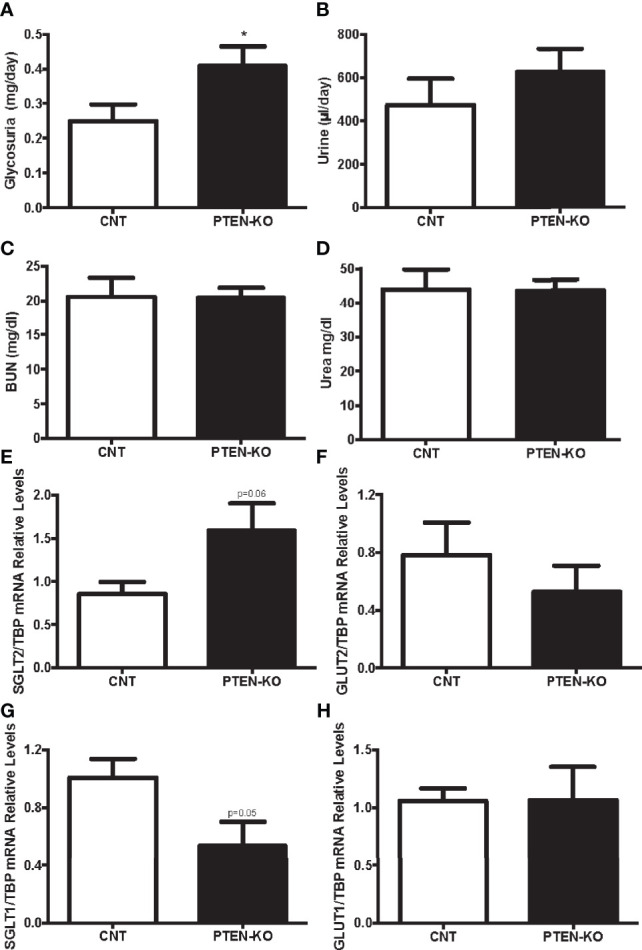
PTEN-KO mice present with modest glycosuria and alterations in proximal tubule kidney glucose transporters. **(A)** Glucose detection in urine. **(B)** Urine excretion in 24 hours. **(C, D)** Blood urea nitrogen (BUN) and urea levels measurements. **(E–H)** Kidney glucose transporters pairs mRNA levels. Data represent the mean ± SEM of 20 mice/group **(A, B)**, 9-10 mice/group **(C, D)** and 6-7 mice/group **(E–H)**. *p < 0.05 *vs*. CNT mice.

As glucose is freely filtered in the renal glomerulus and fully reabsorbed in the proximal tubule, we checked whether PTEN-KO mice had alterations in renal glucose handling. PTEN-KO mice excreted more glucose in urine during 24h than the CNT mice (**Figure 3A**). Accordingly, PTEN-KO mice had a clear tendency to higher urine output than CNT mice (**Figure 3B**). Renal function parameters showed that neither BUN (**Figure 3C**) nor urea (**Figure 3D**) were different between groups, reflecting normal renal function. Sodium-glucose cotransporter 2 (SGLT2), which is located in the apical membrane, had a trend to be increased in PTEN-KO mice compared to CNT mice (**Figure 3E**). Whereas Glucose transporter 2 (GLUT2), which is located in the basolateral membrane, had a trend to be decreased in PTEN-KO mice compared to CNT mice (**Figure 3F**). The second pair of glucose transporters sodium-glucose cotransporter 1/glucose transporter 1 (SGLT1/GLUT1) also showed altered renal expression. Thus, the higher glucose affinity SGLT1 showed borderline significant reduction of its expression in PTEN-KO mice compared to the CNT mice (**Figure 3G**); GLUT1 showed no changes in expression after elimination of PTEN (**Figure 3H**).

### Altered Kidney Glucose Transporters in PTEN-Downregulated HK-2 Cells

**Figure 4:**
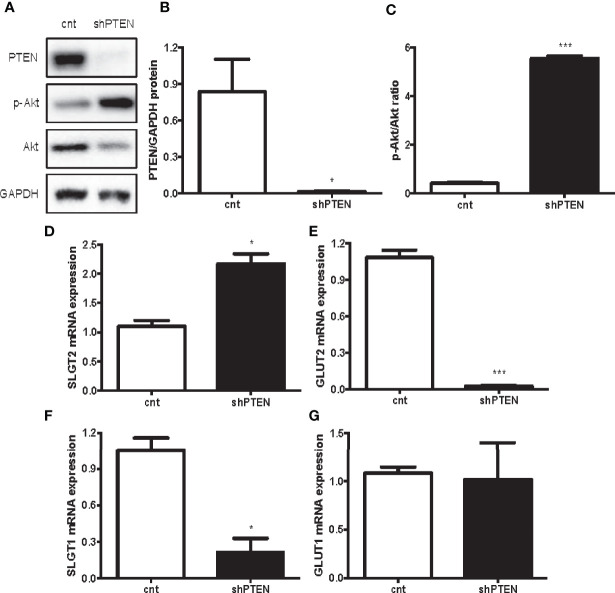
Downregulation of PTEN in HK-2 cells leads to kidney glucose transporters alterations. **(A–C)** Representative Western blot analysis of PTEN, p-AKT and AKT from CNT and shPTEN cells extracts. **(D–G)** Expression of kidney glucose transporters pairs. Data represent the mean ± SEM from 3 independent experiments. *p < 0.05; ***p < 0.001 *vs*. CNT cells.

The results of the *in vitro* experiments with HK-2 cells with downregulation of PTEN show that the intervention successfully decreased PTEN levels (**Figures 4A, B**). Moreover, the ratio p-AKT/AKT showed that PI3K/AKT pathway was strongly activated in shPTEN cells compared to CNT cells (**Figures 4A, C**). Similarly, to the *in vivo* results, the expression of the first pair of glucose transporters (SGLT2/GLUT2) was also altered. SGLT2 was significantly increased in shPTEN cells in comparison to CNT cells (**Figure 4D**). While GLUT2 showed a statistically significant decrease in shPTEN cells compared to CNT cells (**Figure 4E**). The higher glucose affinity SGLT1 showed a statistically significant decrease in expression in shPTEN cells, compared to CNT cells (**Figure 4F**). Finally, GLUT1 was not affected (**Figure 4G**).

### Altered Liver Histopathology in PTEN-KO Mice

**Figure 5:**
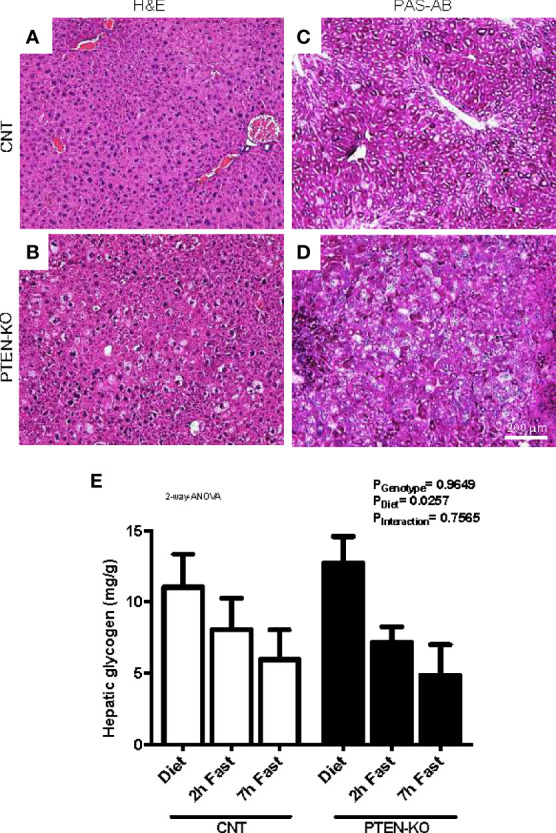
Hepatocellular ballooning and glycogen levels in PTEN-KO mice livers. **(A, B)** Representative H&E-staining of liver sections. **(C, D)** Representative PAS-AB-staining of liver sections. **(E)** Hepatic glycogen levels. Original magnification x40. Data presents representative staining from 3 mice/group **(A–D)** and the mean ± SEM of 11-13 mice/group **(E)**.

The H&E stains from liver of PTEN-KO mice showed an altered morphology of the hepatocytes (**Figures 5A, B**). Hepatocytes were enlarged, cells presented with different cytoplasmic shape and showed signs of inflammation and hepatocellular ballooning (i.e. hepatocyte degeneration/injury). PAS staining suggested that the levels of glycogen in PTEN-KO mice were similar to in CNT mice (**Figures 5C, D**). Measurements of total hepatic glycogen from liver homogenates in animals showed no differences between groups neither in the unfasted nor in the fasted state (**Figure 5E**).

### Delayed Gluconeogenesis in PTEN-KO Mice

**Figure 6:**
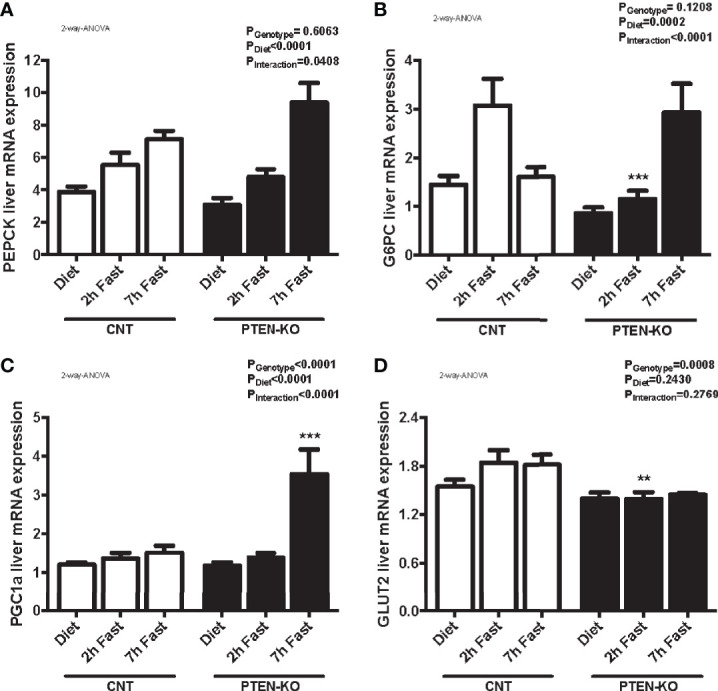
Delayed gluconeogenesis in PTEN-KO mice. Hepatic mRNA levels of PEPCK **(A)**, G6PC **(B)**, PGC1a **(C)**, and GLUT2 **(D)**. Data represent the mean ± SEM of 10-15 mice/group. **p < 0.001; ***p < 0.001 *vs*. CNT mice.

In CNT mice, the expression of PEPCK (**Figure 6A**) and G6PC (**Figure 6B**) were increased after 2 hours of fasting. Levels of G6PC returned to normal after 7 hours of fasting. However, the gluconeogenic genes were not activated until 7h of fasting in PTEN-KO mice (**Figures 6A, B**). PGC1a, a transcriptional coactivator involved in PEPCK and G6PC regulation, was also strongly activated at 7h of fasting in PTEN-KO mice (**Figure 6C**). Finally, GLUT2, which is the hepatic glucose transporter in charge of transferring glucose between hepatocytes and the bloodstream, was lower in the PTEN-KO mice (**Figure 6D**).

### Lipid Metabolism Disorders in PTEN-KO Mice

As fats are used as an energy source in low glucose states, we also focused our research on lipid metabolism. We observed that PTEN-KO mice presented a tendency to have higher levels of circulating β-hydroxybutyrate during meals and after 2h of fasting compared to CNT mice (**Supplementary Figure 3A**). However, when they were in a more prolonged fasting state, they had similar ketone body levels to the control group that was also in starvation. In addition, urine pH was similar between groups, which rules out ketoacidosis (**Supplementary Figure 3B**). Furthermore, after 7 hours of fasting, there were no statistically significant differences in lipid profile between CNT and PTEN-KO mice (Total Cholesterol CNT: 117.8±6.5, PTEN-KO:110.1±6.4; LDL Cholesterol CNT: 16.9±3.2, PTEN-KO: 16±4.4; HDL Cholesterol CNT: 89.3±2.6, PTEN-KO: 83.7±6.4; Triglycerides CNT: 89.3±5.1, PTEN-KO: 83.7±5.9. Data are Mean±SD).

**Figure 7:**
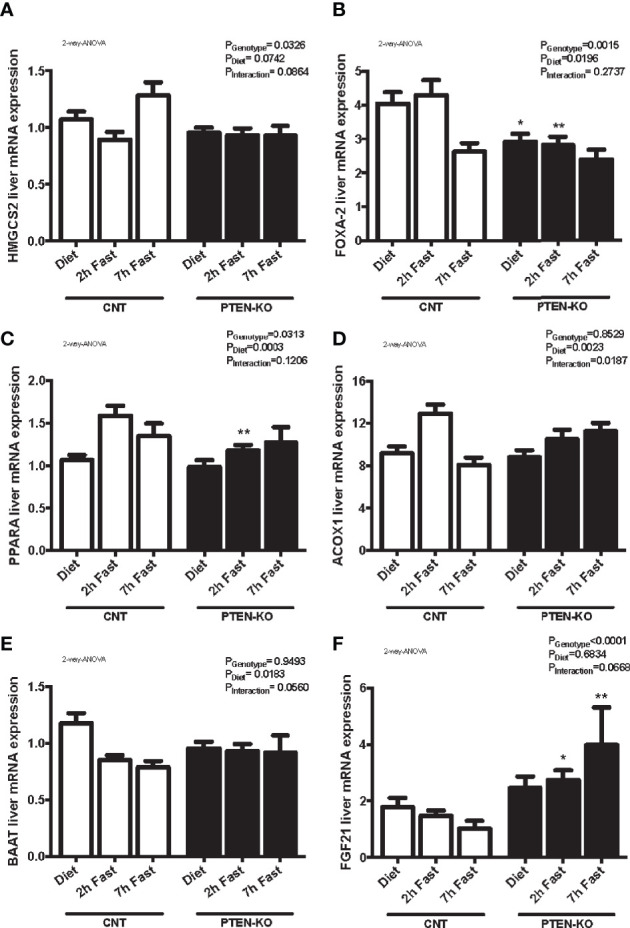
Lipid metabolism alterations in PTEN-KO mice. Hepatic mRNA levels of HMGCS2 **(A)**, FOXA-2 **(B)**, PPARA **(C)**, ACOX1 **(D)**, BAAT **(E)**, and FGF21 **(F)** mRNA levels Data represent the mean ± SEM of 10-15 mice/group. *p < 0.05; **p < 0.001 *vs*. CNT mice.

We analyzed different genes involved in β-oxidation and ketogenesis. We observed that the expression of hydroxymethylglutaryl-CoA synthase 2 (HMGCS2) was only slightly increased at 7 hours of fasting in CNT mice but this was not observed in KO mice (**Figure 7A**). In addition, Forkhead box A2 (FOXA2), was down-regulated after 7h of fasting in CNT mice, while in PTEN-KO mice its expression was already lower in the basal fed state and after 2h fasting (**Figure 7B**). Peroxisome proliferator-activated receptor A gene (PPARA) and its target-gene acyl-CoA oxidase 1 (ACOX1) were upregulated in early fasting in CNT mice, whereas in PTEN-KO mice this induction was delayed (**Figures 7C, D**). Bile acid-CoA:amino acid N-acyltransferase (BAAT), which amidates bile acids as well as long- and very-long-chain acyl-CoAs (C12:0–C26:0) with glycine and taurine, decreased in fasted states in CNT mice, while no reduction was observed in PTEN-KO mice (**Figure 7E**). Lastly, we measured fibroblast growth factor 21 (FGF21), which is a crucial intermediate between hepatic lipid metabolism, diet and PPARA in ketogenic states, and observed that FGF21 mRNA levels increased in PTEN-KO mice, but no increase was observed in CNT mice (**Figure 7F**). Finally, we could not see significant differences between CNT and PTEN-KO mice in the hepatic expression of carnitine palmitoyl transferase 1 (CPT1) (**Supplementary Figure 3C**), which transports fatty acids into mitochondria and is induced by fasting, and angiopoietin-like protein 8 (ANGPTL8) (**Supplementary Figure 3D**), which regulates serum triacylglycerol levels by inhibiting LPL and is repressed by fasting.

## Discussion

In the present paper, we characterize the phenotype of a pan-inducible PTEN-KO mouse. The characteristics of this mouse resemble some of those observed in patients with hypoinsulinemic hypoglycemia, suggesting that it could be a good model to study this condition.

PTEN is the main negative regulator of PI3K/AKT pathway. This pathway has an important role as effector of the insulin actions, like activating glucose uptake and inhibiting lipolysis in adipose tissue, activating glucose uptake and glycogen synthesis in muscle, and inhibiting gluconeogenesis, glycogenolysis and glucose release in the liver. Furthermore, PTEN is also an important tumor suppressor (24). The mutation of the homologue of the PTEN in *C. elegans* (DAF-18), suggests that the activity of this enzyme has longevity-promoting activity related to signaling through insulin-like growth factors (25, 26). Further research in tissue-specific deletion of PTEN yielded interesting results in mouse models. Thus, PTEN suppression in liver and fat improved glycaemia and insulin sensitivity in type 2 diabetes mellitus (T2DM) mice, protecting diabetic mice from developing diabetes (27). Furthermore, in diabetic patients, an association between a PTEN polymorphism and T2DM was observed (28). PTEN functions have been described to be highly dependent on the specific tissue in which it is expressed. In muscle (29), liver (30), pancreas (31) and fat (32) the lack of PTEN affects glucose homeostasis and results in resistance to diabetes. After the organ-specific deletions of PTEN, a PTEN haploinsufficiency (PTEN^+/-^) mouse was generated to analyze the global effect of PTEN deletion on glucose homeostasis showing that those mice had higher insulin sensitivity (33). However, as homozygous PTEN inactivation results in early embryonic lethality, no previous studies have been conducted to assess glucose homeostasis in whole body PTEN^-/-^ mice.

Our results show that animals with full organism PTEN deletion in adulthood present with very low blood glucose levels and hypoinsulinemia, with preserved pancreatic structure and *in vitro* insulin secretion in response to glucose. Our results are in agreement with those of Kinross et al in which mutations that constitutively activate the catalytic subunit of PI3K show also low levels of glucose with concomitant hypoinsulinemia (34). However Nguyen et al. observed an increase of islet mass in a β-cells-specific PTEN deficiency mouse (35). This difference could be explained by the fact that our mice are full KO and PTEN is deleted after animals fully developed, contrary to the data of Nguyen in which specific deletion of PTEN in β-cells was produced from the embryonic state. We also found an altered response to fasting. Thus, control animals did not show modifications of their glucose levels after 7 hours of fasting, contrary to PTEN-KO mice which after starting with lower levels at basal timepoint, showed further decreases after 7 hours. This fact could be explained by an increase in the rate of glucose utilization and/or a decrease in glucose formation. Thus, the *in vivo* GTT and ^3^H-glucose uptake experiments show that animals with the elimination of PTEN have a faster use of exogenously administered glucose. These results are in agreement with those obtained in the animal with specific liver PTEN deletion, which showed decreased fasting glucose levels and improved glucose tolerance (30). Furthermore, the PTT also shows that the peak in glucose observed after pyruvate administration in normal mice is absent in PTEN-KO mice, demonstrating that both the elimination and the formation of glucose by hepatic gluconeogenesis are altered.

Another way mice could achieve low levels of glucose is by losing glucose through urine. Urine is usually glucose-free, as all the glucose filtered in the glomerulus is reabsorbed in the proximal tubule. Renal tubular glucose handling is achieved by two pairs of glucose transporters located in the proximal tubule, SGLT2/GLUT2 that absorb more than 90% of glucose and SGLT1/GLUT1 that absorb the rest (36, 37). The regulation of its expression is not highly understood yet, although it is suggested that independent regulatory mechanisms by insulin and glucose are playing a role (38). In our PTEN-KO mice and HK-2 proximal tubular cells with elimination of PTEN, both GLUT2 and SGLT1 were downregulated. In addition, SGLT2 expression was increased. Insulin signaling has been shown to increase renal tubular SGLT2 expression (39), whereas changes in GLUT2 expression seem to be further regulated by blood glucose levels, as in diabetic mice fasted overnight, the increase of GLUT2 expression in tubular cells was abolished (40). Thus, the findings observed in our experiments in mice and cells with overactivation of the insulin receptor agree with previous results. However, the regulation of the second pair of glucose transporters in renal tubular cells is not so well understood. In any case, a decrease in the expression of the higher glucose affinity SGLT1 has been previously reported in renal proximal tubular cells with overactivation of the PI3K/AKT pathway. Thus, it seems that the increase in urine glucose excretion observed in our mice could partially contribute to the decrease in blood glucose levels. The increase in SGLT2 expression will reabsorb most of the glucose in the early parts of the proximal tubule, although the system will be limited by the high Km for glucose of SGLT2 and because of a potential intracellular saturation of glucose in the cell due to the decrease of GLUT2 transporters. Then, glucose will be delivered to the later portions of the proximal tubule but the decrease in SGLT1 transporters will impede its total reabsorption and contribute to its presence in urine. We should point out that the glucose filtered in the glomerulus is dependent on the glucose concentration in plasma and, therefore, in the animals with full PTEN deletion is likely very low. However, the deregulation of glucose reabsorption in the proximal tubule might cause very high levels of glucosuria if normal levels of glucose were present in blood.

One more possible explanation for the increased glucose consumption in PTEN KO animals is the apparition of tumors with high glucose demands. The elimination of PTEN in adulthood with the same method has been shown to produce tumors in the colon, thyroid gland, prostate and endometrium 9 to 12 weeks after tamoxifen administration (18, 41, 42). However, after modifying the background of the animals by crossing with C57BL6/129S1Sv/129X1SvJ/SJL mice, we did not observe evident macroscopic tumors in the necropsies. Furthermore, animals were sacrificed 6 weeks after the injection of tamoxifen. In any case, we can not rule out that part of the low glucose levels observed in the animals was due to microtumors with high glucose uptake although the ^3^H-glucose uptake experiments did not show any specific tissue with a higher glucose uptake than the rest.

As the results of PTT suggested suppression in gluconeogenesis due to enhanced hepatic insulin sensitivity, we focused on the liver as the main organ contributing to this process. Indeed, it has been previously shown that overactivation of the PI3K activity by mutations in its regulatory subunit *in vivo* increased insulin sensitivity and induced hypoglycemia (43). Furthermore, specific liver deletion of the catalytic subunit of PI3K leads to reduced insulin signaling and increased gluconeogenesis (44). Mice with liver-specific deletion of PTEN can also develop liver steatosis accompanied by low body fat and increased glycogen synthesis (30). However, in our mice, we did not detect a higher accumulation of glycogen. Again, this disagreement with previous results could be explained by the elimination of PTEN in the full animal, contrary to the studies of Stiles et al, in which the deletion was liver-specific (30). However, we did observe that PTEN-KO mice presented hepatocellular ballooning, which is a histological parameter used in the diagnosis and grading of nonalcoholic fatty liver disease and considered to be the result of hepatocyte injury by several factors, including lipotoxicity. In addition, we observed the effects of PTEN elimination in genes related to gluconeogenesis. Under fasting conditions, hepatic glucose production is critical as a source to maintain the basic functions in other tissues. The reduced levels of G6PC and PEPCK in PTEN-KO mice after two hours of fasting are indicative of a lack of activation of gluconeogenesis, partly explaining why PTEN-KO mice were unable to maintain normal glucose levels. This activation occurred very late compared with CNT mice. Thus, the expression of PGC1a, a gene involved in the activation of PEPCK and G6PC (45), was only increased after 7 hours of fasting in PTEN-KO mice, demonstrating that gluconeogenesis is only activated after very severe low glucose levels. This can be explained by the fact that constitutive activation of AKT in PTEN-KO promotes, either dependent or independent of the insulin receptor, the inhibition of CBP/CREB and FOXO1 thus preventing the activation of PGC1α, PEPCK and G6PC (46, 47). These results reflect delayed gluconeogenesis in PTEN-KO mice and support a stronger effect of insulin signaling than of low glucose levels until a critically low glucose threshold level is achieved. Thus, after that threshold is achieved, the secretion of adrenal cortisol could increase, promoting transcriptional activation of hepatic gluconeogenic genes (48). Then, this mice model corroborates an insulin inhibitory effect in gluconeogenesis, agreeing with the results of Stiles et al and Peyrou et al which showed reduced levels of G6PC and PEPCK in the model with liver-specific deletion of PTEN (30, 49). However, we found more exacerbated low glucose levels in our mice, probably as a consequence of the deletion of PTEN and the activation of the PI3K/AKT pathway in other organs as inhibition of hepatic gluconeogenesis in the liver by signals from adipose tissue, pancreas or hypothalamus has also been shown (50). On the other hand, the expression GLUT2, which facilitates the final step in the transport of glucose out of the liver and into the bloodstream, is diminished in PTEN-KO mice. The role of GLUT2 on gluconeogenesis is not clear. However, our results agree with those of Lamia et al. (51) in which fasting low glucose levels induced by liver-specific knockdown of Bmal1 were attributable to a marked decrease in the expression of GLUT2.

Liver-specific PTEN deletion has also been shown to alter hepatic lipid metabolism. Thus, and in agreement with our results, animals showed signs of steatosis with no changes in plasma triglycerides (30, 49). These results can be explained because the hyperactivation of AKT stimulates the expression of the lipogenic transcription factor sterol regulatory element-binding transcription factor (SREBP) 1c (52). Moreover, AKT inactivates FOXO1, a repressor of SREBP1c transcription (53). Thus, PTEN deletion will result in upregulation of SREBP1c and its downstream targets (e.g. fatty acid synthase) and induction of *de novo* lipogenesis in hepatocytes (30). However, the regulation of other lipid key pathways such as fatty-acid β-oxidation and ketogenesis have not been investigated in PTEN-KO mice, particularly under fasting conditions. In our full PTEN-KO, we tested ketone body production as it is a critical metabolic fuel in a prolonged fasting state. The results indicated that ketone body levels in PTEN-KO mice were similar to those in CNT animals. Accordingly, we did not find an increase in the expression of a key ketogenic gene, the HMGCS2 gene, which catalyzes the first reaction of the ketogenesis pathway in the mitochondria (54). Regarding the expression of genes involved in fatty acid β-oxidation, we observed a delayed or even absent response to fasting, suggesting that the lack of PTEN not only altered glucose metabolism but also slowed down the metabolism of lipids. Thus, FOXA2, which activates transcription of genes related to β-oxidation and ketogenesis (55), was downregulated in CNT animals after 7h of fasting, while in PTEN-KO mice it was lower in the basal time point and was not modified during fasting. We also studied PPARA that triggers the transcription of genes related to β-oxidation and fatty acid transport (56–59). PPARA gene was activated in early fasting timepoints in CNT mice, whereas in PTEN-KO mice there was a delayed induction. Accordingly, the PPARA target-gene ACOX1, which participates in peroxisomal β-oxidation (60), showed the same profile. Lastly, we measured FGF21, which is a crucial intermediate between hepatic lipid metabolism, diet and PPARA in ketonic states, and observed that FGF21 levels were increased in PTEN-KO mice in contrast to CNT mice in which expression levels were unmodified by fasting. FGF21 is an adaptive response hormone to the late stages of starvation, when it may regulate the utilization of fuel derived from tissue breakdown (61). Thus, the increase in FGF21 expression in PTEN-KO mice underscores the higher impact of metabolic energy restriction in these mice.

Altogether, our results demonstrate that pan-inducible PTEN-KO mice are a good model to study the metabolic interactions between glycidic and lipidic metabolism in hypoinsulinemic hypoglycemia, and the possible targeting of PTEN as a mediator in the disease. Furthermore, this model shows additional characteristics compared to the liver-specific KO mice as it takes into account metabolic effects of PTEN in all the related tissues, like liver, kidney, muscle, and fat.

## Data Availability Statement

The original contributions presented in the study are included in the article/**Supplementary Material**. Further inquiries can be directed to the corresponding author.

## Ethics Statement

The animal study was reviewed and approved by University of Lleida.

## Author Contributions

MC-M, RJ, and JV conceived and designed the experiments. MC-M, AP-G, CG, SR, and ND performed the experiments. AG-C provided technical support. MC-M, RJ, and JV analyzed the date. RJ and JV obtained funding. MC-M and JV wrote the paper. All authors contributed to the article and approved the submitted version.

## Funding

This work was supported by grants from Instituto de Salut Carlos III PI15/00960, PI17/01089 and PI18/610, (co-funded by European Regional Development Fund “A way to achieve Europe”). MC-M was supported by the *Secretaria d’Universitats i Recerca del Departament d’Economia i Coneixement de la Generalitat de Catalunya*, by FSE (EU) funds and by IRBLleida grant supported by *Diputació de Lleida* and *Gerència Territorial de l’Institut Català de la Salut a Lleida, Alt Pirineu i Aran, i Gestió de Serveis Sanitaris*.

## Conflict of Interest

The authors declare that the research was conducted in the absence of any commercial or financial relationships that could be construed as a potential conflict of interest.

## Publisher’s Note

All claims expressed in this article are solely those of the authors and do not necessarily represent those of their affiliated organizations, or those of the publisher, the editors and the reviewers. Any product that may be evaluated in this article, or claim that may be made by its manufacturer, is not guaranteed or endorsed by the publisher.
