# Supplementary material for: PTEN Deletion in Adult Mice Induces Hypoinsulinemia With Concomitant Low Glucose Levels
Source: Front Endocrinol (Lausanne). 2022 Feb 25;13:850214. doi: 10.3389/fendo.2022.850214 (PMC8914015; doi:10.3389/fendo.2022.850214)
Supplement: Supplementary file 1 [file DataSheet_1.zip › Supplementary Figure Legends.DOCX]

**Supplementary figure legends**

**Supplementary Figure 1. PTEN-KO mouse generation.** (A) Breeding scheme. (B) PCR analysis to detect the deleted PTEN band in different random organs (from left to right: thyroid gland, uterus or prostate and pancreas). (B,C) Representative Western blot analysis of PTEN, p-AKT and AKT from CNT mice and PTEN-KO mice extracts normalized to GAPDH from liver. Data presents the mean ± SEM of 12 mice/group *p<0.05; **p<0.001; *** p<0.001 vs. CNT mice.

**Supplementary Figure 2. General mouse monitoring measurements.** (A) Food intake in a day. (B) Water intake in a day. (C) Weight at 2 months after tamoxifen injection, before the sacrifice. (D) Body fat at 2 months after tamoxifen injection, before the sacrifice. Data presents the mean ± SEM of 14-18 mice/group (A,B), 5-10 mice/group (C) and 7 mice/group (D). *** p<0.001 vs. CNT mice.

**Supplementary Figure 3. PTEN-KO mice do not have defects neither in ketone bodies formation nor in the hepatic expression of two lipid metabolism genes (CPT1 and ANGPTL8).** (A) β-hydroxybutyrate serum levels were normally induced in PTEN-KO. (B) Urine pH measurement rules out ketoacidosis IN PTEN-KO. (C,D) Liver (C) CPT1 and (D) ANGPTL8 mRNA levels were determined by real time qRT-PCR and normalized to the mean of TBP and Ppia expression. Data presents the mean ± SEM of 10-15 mice/group (A,B,C,D)
