# Supplementary material for: PTEN Deletion in Adult Mice Induces Hypoinsulinemia With Concomitant Low Glucose Levels
Source: Front Endocrinol (Lausanne). 2022 Feb 25;13:850214. doi: 10.3389/fendo.2022.850214 (PMC8914015; doi:10.3389/fendo.2022.850214)
Supplement: Supplementary file 1 [file DataSheet_1.zip › Supplementary Tables.DOCX]

**Supplementary tables**

**Supplementary Table 1. Real time qRT-PCR primers.**

| **Gene** | **Sequences** | **bp** | **ID** |
| --- | --- | --- | --- |
| **Ppia** | FWD: AGACTGAATGGCTGGATGG | 128 | [NM_008907.2](https://www.ncbi.nlm.nih.gov/entrez/viewer.fcgi?db=nucleotide&id=1418589268) |
|  | REV: GTCGGAAATGGTGATCTTCTTG |  |  |
| **Tbp** | FWD: AAA ATG GTG TGC ACA GGA GCC | 141 | [NM_013684.3](https://www.ncbi.nlm.nih.gov/entrez/viewer.fcgi?db=nucleotide&id=172073170) |
|  | REV: CAC ATC ACA GCT CCC CAC CAT |  |  |
| **mHMGCS2** | FWD: AGACTCCCGGAGACGCATGT | 239 | [NM_008256.4](https://www.ncbi.nlm.nih.gov/entrez/viewer.fcgi?db=nucleotide&id=239937452) |
|  | REV: GTTTGGGTAGCAGCTCGGCTCA |  |  |
| **mFoxa2** | FWD: AGTATGCTGGGAGCCGTGAAG | 218 | NM_001291065.1 |
|  | REV: CCAGCGCCCACATAGGAT |  | NM_010446.3 |
| **mPEPCK** | FWD: CCAGTGCCCCATTATTGAC | 249 | NM_011044.3 |
|  | REV: CCGAAGTTGTAGCCGAAGAA |  |  |
| **mG6PC** | FWD: ACTTTCCCCACCAGGTCGT | 169 | [NM_008061.4](https://www.ncbi.nlm.nih.gov/entrez/viewer.fcgi?db=nucleotide&id=927442691) |
|  | REV: ACCCCTAGCCCTTTTAGTAGCA |  |  |
| **mPGC1a** | FWD: GATCACGTTCAAGATCGCCCTAC | 178 | NM_008904.2 |
|  | REV: TAAATCACACGGCGCTCTTC |  |  |
| **mPPARa1** | FWD: ATTTGGGCGTATCTCACCG | 248 | [NM_011144.6](https://www.ncbi.nlm.nih.gov/entrez/viewer.fcgi?db=nucleotide&id=164663878) |
|  | REV: GGACTTTCCAGGTCATCTGC |  |  |
| **mCPT1** | FWD: ACATCGTGAGTGGCGTCCT | 171 | [NM_013495.2](https://www.ncbi.nlm.nih.gov/entrez/viewer.fcgi?db=nucleotide&id=162287141) |
|  | REV: GACCCGAGAAGACCTTGACCATA |  |  |
| **mAcox1** | FWD: TAACTTCCTCACTCGAAGCCA | 283 | [NM_015729.3](https://www.ncbi.nlm.nih.gov/entrez/viewer.fcgi?db=nucleotide&id=429484482) |
|  | REV: AGTTCCATGACCCATCTCTGTC |  |  |
| **mGLUT2** | FWD: TTGTCATCGCCCTCTGCT | 228 | [NM_031197.2](https://www.ncbi.nlm.nih.gov/entrez/viewer.fcgi?db=nucleotide&id=165377236) |
|  | REV: CACTCTCTGAAGACGCCAGGAA |  |  |
| **mANGPTL8** | FWD: GCCTGTCGGAGATTCAGGTG | 225 | [NM_001080940.1](https://www.ncbi.nlm.nih.gov/entrez/viewer.fcgi?db=nucleotide&id=124301203) |
|  | REV: GGCCAGTGAGAGCCCATAAG |  |  |
| **mFGF21** | FWD: TGAAGCCAGGGGTCATTCAA | 203 | [NM_020013.4](https://www.ncbi.nlm.nih.gov/entrez/viewer.fcgi?db=nucleotide&id=146134956) |
|  | REV: GTTTGGGGAGTCCTTCTGAGG |  |  |
| **mBAAT** | FWD: TGGCTTACTGGAACTATGATG | 188 | [NM_007519.3](https://www.ncbi.nlm.nih.gov/entrez/viewer.fcgi?db=nucleotide&id=211904119) |
|  | REV: GGCTCTTATTTGTTTTAGGTTA |  |  |
| **SGLT1** | Mm00451203_m1 (Applied Biosystems) | 109 | [NM_019810.4](http://www.ncbi.nlm.nih.gov/nuccore/NM_019810.4) |
|  | Hs01573790_m1 (Applied Biosystems) | 78 | [NM_000343.3](http://www.ncbi.nlm.nih.gov/nuccore/NM_000343.3) |
| **SLGT2** | Mm00453831_m1 (Applied Biosystems) | 79 | [NM_133254.3](http://www.ncbi.nlm.nih.gov/nuccore/NM_133254.3) |
|  | Hs00894642_m1 (Applied Biosystems) | 75 | [NM_003041.3](http://www.ncbi.nlm.nih.gov/nuccore/NM_003041.3) |
| **GLUT1** | Mm01192270_m1 (Applied Biosystems) | 75 | [NM_011400.3](http://www.ncbi.nlm.nih.gov/nuccore/NM_011400.3) |
|  | Hs00892681_m1 (Applied Biosystems) | 76 | [NM_006516.2](http://www.ncbi.nlm.nih.gov/nuccore/NM_006516.2) |
| **GLUT2** | Mm00446229_m1 (Applied Biosystems) | 61 | [NM_031197.2](http://www.ncbi.nlm.nih.gov/nuccore/NM_031197.2) |
|  | Hs01096908_m1 (Applied Biosystems) | 65 | [NM_000340.1](http://www.ncbi.nlm.nih.gov/nuccore/NM_000340.1) |

**Supplementary Table 2. Western blot primary and secondary antibodies.**

| **Protein** | **Company** | **Cat. Nº** | **Host** | **Reactivity** | **Dilution** |
| --- | --- | --- | --- | --- | --- |
| **PTEN** | Cell Signaling | 9188 | Rabbit | H,M,R | 1/1000 |
| **pAKT** | Cell Signaling | 4060S | Rabbit | H,M,R | 1/1000 |
| **AKT** | Santa Cruz | sc-5298 | Mouse | H,M,R | 1/1000 |
| **GAPDH** | BioLegent | 919501 | Mouse | H,M,R | 1/1000 |
| **Anti-mouse IgG-HRP** | Jackson Immuno Research | 115-035-003 | Goat | Secondary antibody | 1/10.000 |
| **Anti-rabbit IgG-HRP** | Cell Signaling | 7074 | Goat | Secondary antibody | 1/10.000 |

**Supplementary Table 3. PCR oligonucleotides.**

| **Gene** | **Sequence** |
| --- | --- |
| **PTEN** | FWD: CAAGCACTCTGCGAACTGAG |
|  | REV: AAGTTTTTGAAGGCAAGATGC |
| **Cre-ER^TM^** | FWD: ACGAACCTGGTCGAAATCAGTGCG |
|  | REV: CGGTCGATGCAACGAGTGATGAG |
| **∆PTEN excised** | FWD: ACTCAAGGCAGGGATGAGC |
|  | REV1: AATCTAGGGCCTCTTGTGCC |
|  | REV2: GCTTGATATCGAATTCCTGCAGC |
