# Supplementary figures and images for: PTEN Deletion in Adult Mice Induces Hypoinsulinemia With Concomitant Low Glucose Levels
Source: Front Endocrinol (Lausanne). 2022 Feb 25;13:850214. doi: 10.3389/fendo.2022.850214 (PMC8914015; doi:10.3389/fendo.2022.850214)

# Supplementary Figure 1

A

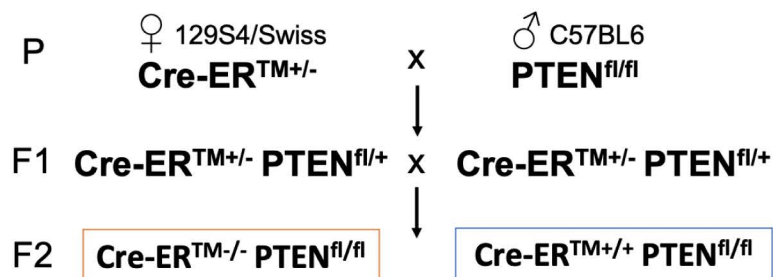

B

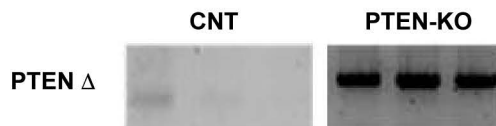

C

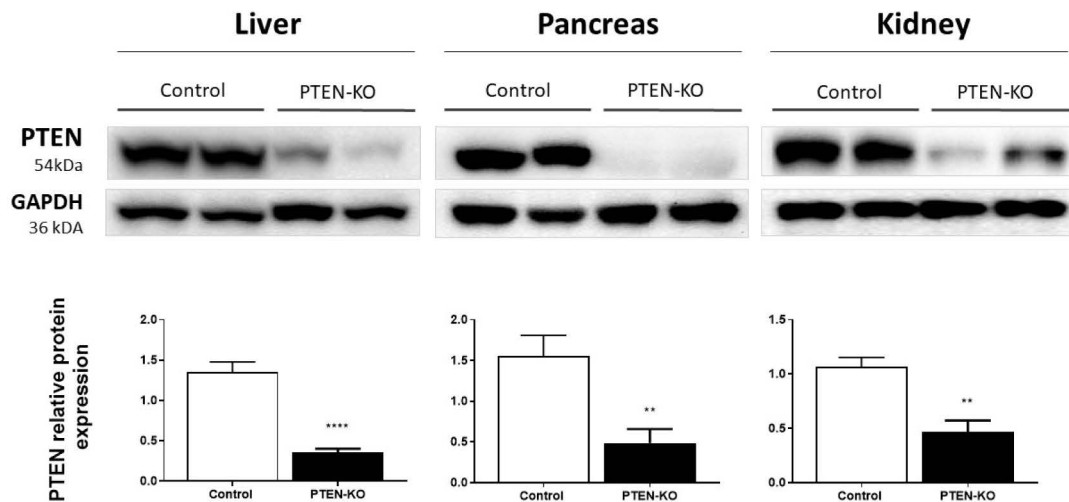

Supplement: Supplementary file 1 [file DataSheet_1.zip › Supplementary Figure 1.PDF]

# Supplementary Figure 2

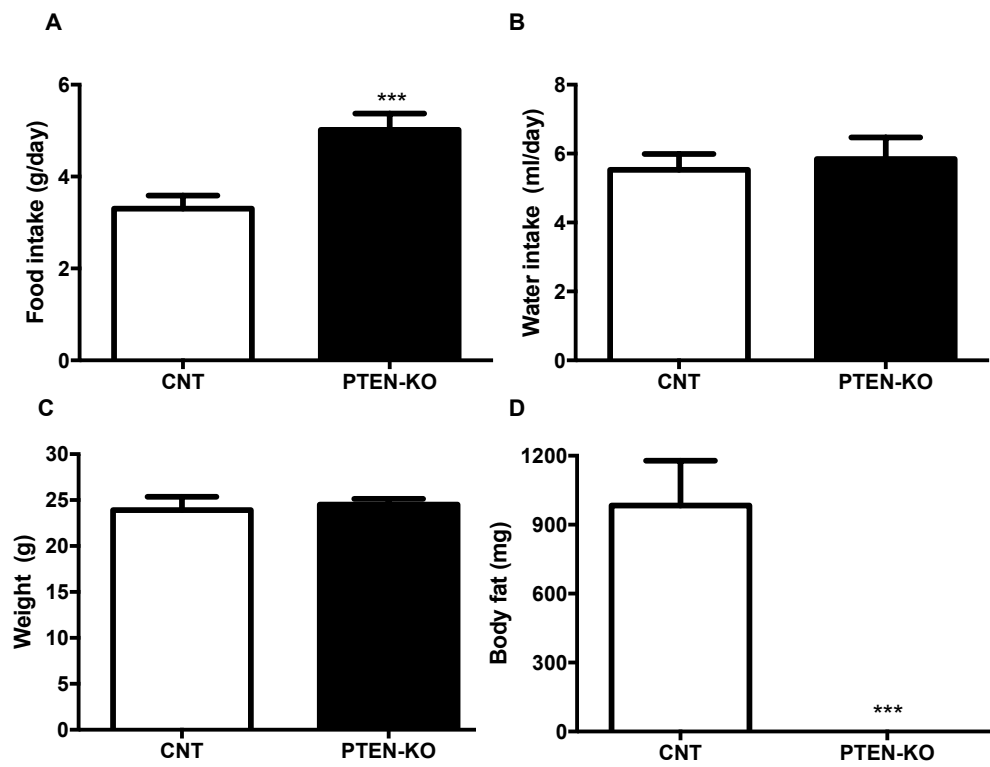

Supplement: Supplementary file 1 [file DataSheet_1.zip › Supplementary Figure 2.PDF]

# Supplementary Figure 3

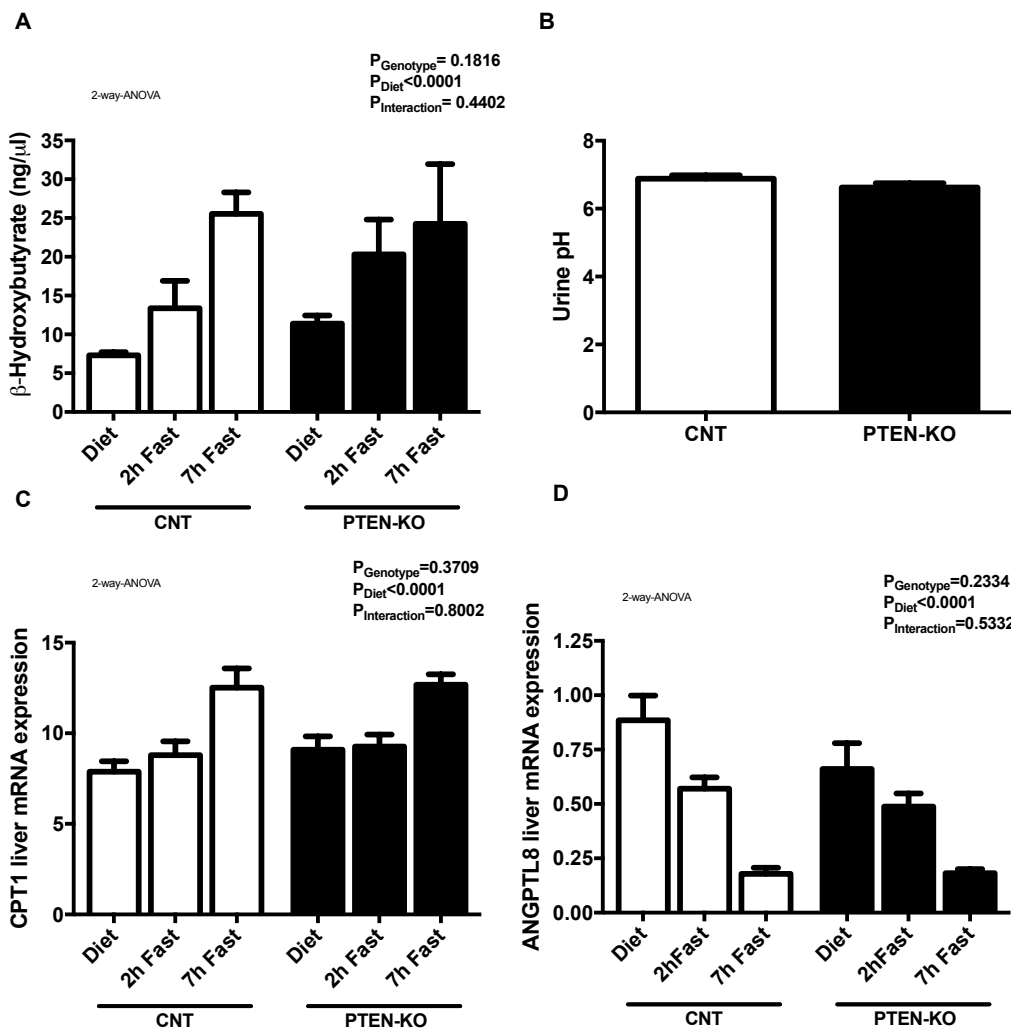

Supplement: Supplementary file 1 [file DataSheet_1.zip › Supplementary Figure 3.PDF]
